# Conformational Barrier of CheY3 and Inability of CheY4 to Bind FliM Control the Flagellar Motor Action in *Vibrio cholerae*

**DOI:** 10.1371/journal.pone.0073923

**Published:** 2013-09-16

**Authors:** Maitree Biswas, Sanjay Dey, Susmita Khamrui, Udayaditya Sen, Jhimli Dasgupta

**Affiliations:** 1 Department of Biotechnology, St. Xavier’s College, Kolkata, India; 2 Crystallography and Molecular Biology Division, Saha Institute of Nuclear Physics, Kolkata, India; National Research Council of Italy, Italy

## Abstract

*Vibrio cholerae* contains multiple copies of chemotaxis response regulator (*Vc*CheY1–*Vc*CheY4) whose functions are elusive yet. Although previous studies suggested that only *Vc*CheY3 directly switches the flagellar rotation, the involvement of *Vc*CheY4 in chemotaxis could not be ruled out. None of these studies, however, focused on the structure, mechanism of activation or molecular basis of FliM binding of the *Vc*CheYs. From the crystal structures of Ca^2+^ and Mg^2+^ bound *Vc*CheY3 we proposed the presence of a conformational barrier composed of the hydrophobic packing of W61, M88 and V106 and a unique hydrogen bond between T90 and Q97 in *Vc*CheY3. Lesser fluorescence quenching and higher *K_m_* value of *Vc*CheY3, compared to its mutants *Vc*CheY3-Q97A and *Vc*CheY3-Q97A/E100A supported our proposition. Furthermore, aforesaid biochemical data, in conjunction with the structure of *Vc*CheY3-Q97A, indicated that the coupling of T90 and Q97 restricts the movement of T90 toward the active site reducing the stabilization of the bound phosphate and effectively promoting autodephosphorylation of *Vc*CheY3. The structure of BeF_3_^−^ activated *Vc*CheY3 insisted us to argue that elevated temperature and/or adequacy of phosphate pool might break the barrier of the free-state *Vc*CheY3 and the conformational changes, required for FliM binding, occur upon phosphorylation. Structure of *Vc*CheY4 has been solved in the free and sulfated states. *Vc*CheY4^sulf^, containing a bound sulfate at the active site, appears to be more compact and stable with a longer α4 helix, shorter β4α4 loop and hydrogen bond between T82 and the sulfate compared to *Vc*CheY4^free^. While pull down assay of *Vc*CheYs with *Vc*FliM_NM_ showed that only activated *Vc*CheY3 can interact with *Vc*FliM_NM_ and *Vc*CheY4 cannot, a knowledge based docking explained the molecular mechanism of the interactions between *Vc*CheY3 and *Vc*FliM and identified the limitations of *Vc*CheY4 to interact with *Vc*FliM even in its phosphorylated state.

## Introduction

*Vibrio cholerae*, the highly motile gram-negative bacterial pathogen that causes cholera, uses chemotaxis and motility to travel to its preferred intestinal niche to colonize [1]. Extensive studies on chemotaxis of *Escherichia coli* or *Salmonella typhimurium* showed that the ligand induced conformational change in methyl accepting chemotaxis protein (MCP) is sensed by the CheA–CheW complex eventually resulting autophosphorylation of the kinase CheA. Autophosphorylated CheA then donates phosphate to the response regulator CheY. Phosphorylated CheY interacts with the flagellar motor protein FliM and influence the direction of flagellar rotation from counter clock wise (CCW) to clock wise (CW) [2], [3]. CCW rotation results smooth swimming and CW rotation causes the cell to tumble [4]. Because of the presence of a single polar flagellum, *V. cholerae* does not tumble as such but reverses direction briefly, allowing the bacterium to randomly reorient itself and swim in a new direction.

The genomes of a large number of bacterial species, including *Vibrio cholerae*, *Pseudomonas aeruginosa*, *Rhodobacter spaeroides*, *Myxococcus xanthus*, *Borrelia burgdorferi*, and *Yersinia pestis*, encode for multiple paralogues of the various chemotaxis genes and chemotaxis in these bacteria is more complex [5], [6]. A recent genomic and bioinformatic analysis of over 450 bacteria indicates that more than 50% of the chemotaxis gene homologs have more than one copy of chemotaxis genes [5] and these genes are involved not only in flagellum-mediated chemotaxis but also in type-4 pilus-based motility [7], [8], polysaccharide biosynthesis associated with pilus-based gliding motility [9] and flagellar morphogenesis [10]. In many cases, however, genetic analysis has not been successful in deciphering the function of these chemotaxis gene homologs [5], [11].

The genome sequence of *V. cholerae* has three sets of Che protein and 45 MCP-like proteins [12]. Each set of *che* genes forms clusters where *che* cluster I (located on chromosome I) contains *cheY1, cheA1, cheY2, cheR1, cheB1* and the putative gene *cheW*; cluster II of chromosome I contains *cheW1, cheB2, cheA2, cheZ* and *cheY3*, while cluster III of chromosome II contains *cheB3, cheD, cheR3, cheW2, cheW3, cheA3 and cheY4*.

**Figure 1:**
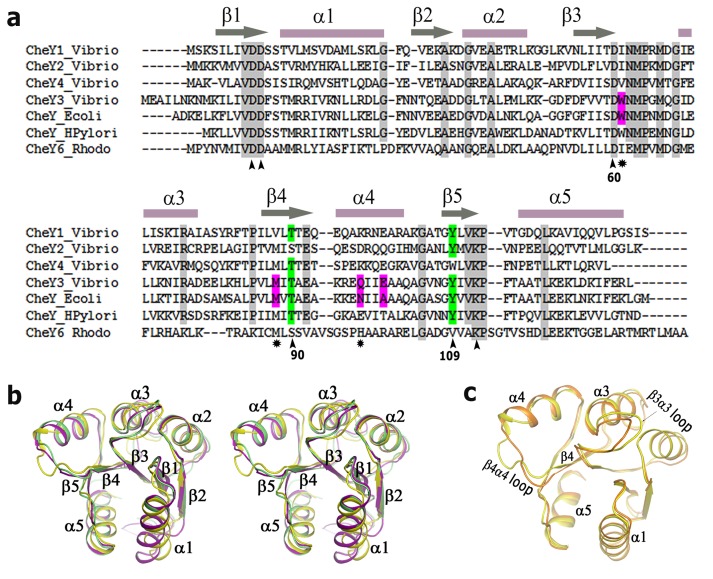
Sequence alignment and overall structure comparison of CheYs. (a) Amino acid sequences of *Vc*CheY1–*Vc*CheY4 are aligned with CheY6 of *Rhodobacter spaeroides,* CheY of *Escherichia coli* and CheY1 of *Helicobacter pylori*. Secondary structural elements are marked and labelled at the top. At the bottom important conserved residues implicated in activation/metal binding are marked as (^∧^) whereas other important residues are indicated as (*); as *Ec*Chey and *St*CheY possess 99% sequence identity only *Ec*CheY was shown in the alignment file. (b) Stereo representation showing the comparison of the overall structures of *Vc*CheY3 (violet), *St*CheY (green), each in free state, with *Vc*CheY4^free^ (yellow); (c) Superposition of the overall structure of *Vc*CheY4^free^ (yellow) on *Vc*CheY4^sulf^ (orange) showing the significant differences in helix α4 and β4α4 loop.

So far, the molecular characterization of all four CheYs of *V. cholerae* (namely, *Vc*CheY1–*Vc*CheY4) is restricted to a few *in vivo* studies where some of the chemotaxis related genes are found to be involved in the virulence of *V. Cholerae* [13]–[15] [3]. Attempts to identify the *V. cholerae cheY* responsible for the flagellar motion showed that a deletion of *cheY*3 impairs chemotaxis [1] while insertional disruption and duplication of the *cheY*4 gene result in decreased and increased motility respectively [13]. Swarming assay and assessment of the swimming behaviour indicated that only *Vc*CheY3 directly switches flagellar rotation, although this study could not rule out the involvement of *Vc*CheY4 in the motor action [14]. Later, Bandyopadhaya and Chaudhuri (2009) showed that inactivation of *cheY3* or *cheY4* generates a less motile and less adherent mutant [15]. Sequence analysis of *Vc*CheYs indicate that only 17% residues are identical among them which comprise the residues involved in binding of the divalent metal ion and stabilization of the phosphorylated intermediate (Figure 1a). This implies that the basic machinery for the phosphorylation is intact for all four *Vc*CheYs. Available literature, however, suggest that deletion of the *cheY1* and *cheY2* genes does not cause any defect in chemotaxis [14] and motility or adherence remains unaffected for the insertional mutants of *cheY1* or *cheY2* [15]. All these observations point to the fact that *Vc*CheY3 and *Vc*CheY4 are the key response regulators to control chemotaxis in *V. cholerae.*

Structures of CheY from different bacterial sources suggest that although all of these response regulators possess an overall (β/α)_5_ fold, small differences in the amino acid sequence or point mutations lead to the subtle conformational variations that make each of these proteins unique in terms of their function [16]–[18]. Also, T87I and T87I/Y106W mutants of *Ec*CheY were found to be phosphorylatable although these mutants were unable to generate clockwise rotation of the flagella [19]. In addition, both of these mutants had ∼5-fold lower autodephosphorylation rates and the mutants were completely resistant to CheZ activity, indicating that an isoleucyl side-chain at position 87 renders *Ec*CheY unable to perform its chemotactic functions [20].

*Vc*CheY3 bears only 37% sequence identity with that of *Vc*CheY4 (Figure 1a) and so far, nothing is known about the structure, mechanism of activation or molecular basis of FliM binding for these two key response regulators, implicated in chemotaxis and virulence of *V. cholerae*. Here we report, the structures of *Vc*CheY3 in Ca^2+^ and Mg^2+^ bound states, BeF_3_^−^ activated *Vc*CheY3 (*Vc*CheY3-BeF_3_^−^) and of the mutant *Vc*CheY3-Q97A. Our structural observations identified a unique conformational barrier in *Vc*CheY3 that controls its phosphorylation event. Implication of this barrier is established by fluorescence spectroscopic study on *Vc*CheY3 and its mutants *Vc*CheY3-Q97A, *Vc*CheY3-Q97A/E100A and *Vc*CheY3-D60A, comparison of their *K_m_* values and pull down assay with *Vc*FliM_NM_. We have also reported the structures of *Vc*CheY4 in free and sulfate bound states here and comparison of these structures helped us to argue that *Vc*CheY4 has a strong tendency to be phosphorylated and the phosphorylated state would be more stable compared to its free state. While our pull down assay showed that only activated *Vc*CheY3 can interact with *Vc*FliM_NM_ and *Vc*CheY4 cannot, structure based docking explained the molecular mechanism of the interactions between *Vc*CheY3 and *Vc*FliM and identified the structural limitations of *Vc*CheY4 to interact with *Vc*FliM even in its phosphorylated state.

## Materials and Methods

### Cloning, Overexpression and Purification

*Vc*CheY3 and *Vc*CheY4 were purified according to the previously described protocols [21], [22]. Briefly, the genes encoding *Vc*CheY3 and *Vc*CheY4 were amplified from *V. cholerae* O395 genomic DNA and cloned into pET28a^+^ vector. After transformation, cells were grown at 37°C until the optical density at 600 nm (OD_600_) reached 0.4 to 0.6. Protein expression was induced by the addition of IPTG (isopropyl-D-thiogalactopyranoside) to a final concentration of 0.1 mM. The cells were harvested by centrifugation and the resuspended pellet was lysed by sonication in presence of PMSF. The cell lysate was then centrifuged (12000 g for 50 mins) at 4°C. The 6×His tagged protein was isolated from the supernatant using Ni^2+^–NTA affinity chromatography (Qiagen) and were eluted with lysis buffer containing 150 mM imidazole. The eluted fractions were checked by 15% SDS–PAGE, pooled and dialyzed overnight against the thrombin clevage buffer (0.05 M Tris–HCl pH 8.0, 150 mM NaCl) and the 6×His tag was cleaved with 1 U thrombin by overnight incubation at 4°C. The proteins were further purified by gel filtration chromatography using a Sephacryl S-100 (GE-Healthcare) column (78×1.4 cm) pre-equilibrated with thrombin cleavage buffer containing 0.02% sodium azide at 4°C.

The gene encoding FliM_NM_ (residue 1–250) was amplified from *V. cholerae* O395 genomic DNA and cloned into pET21b^+^ vector with a C-terminal 6×His-tag to get optimal expression level and solubility. The FliM_NM_ protein was purified by growing cells in LB media to an optimal density 0.6–0.8 at 600 nm and induced with 1 mM IPTG. The cells were harvested after induction at 37°C for 3 h. Cell pellet was resuspended in lysis buffer containing 50 mM Tris-HCl pH 7.5, 250 mM NaCl, 1 mM PMSF and 10 mg lysozyme and lysed by sonication. After centrifugation (14000×g, for 45 mins and at 4°C) FliM_NM_ with C-terminal 6×His-tag was isolated from the supernatant by using Ni^2+^-NTA agarose (Qiagen) and the protein was eluted with lysis buffer containing 200 mM Imidazole. After checking in 12% SDS-PAGE the eluted fractions were dialyzed against the lysis buffer.

### Mutagenesis

*Vc*CheY3-D60A, *Vc*CheY3-Q97A and *Vc*CheY3-Q97A/E100A were prepared by two-step PCR and verified by commercial sequencing. All the mutant proteins were purified using the same protocol described for the wild type protein.

### FliM_NM_-CheY Interaction through Nickel Pull-down Assay

50 µl of Ni^2+^-NTA slurry (Qiagen) was washed three times with binding buffer containing 10 mM imidazole, 150 mM NaCl, 5 mM MgCl_2_, 0.15% Tween 20 and 50 mM Tris-Cl (pH 7.5) and the resin was then incubated with 0.1 ml purified FliM_NM_-His protein in a concentration of 0.2 mg/ml at 25°C for 20 mins with gentle shaking. The beads were then washed for three times with the binding buffer before adding *Vc*CheY3, *Vc*CheY3-Q97A, *Vc*CheY3-Q97A/E100A, *Vc*CheY3-D60A or *Vc*CheY4. For activation, respective protein was pre-incubated for 20 mins with BeF_3_- (100 mM). The mixture was then added in the FliM_NM_-His bound Ni^2+^-NTA resin maintaining 1∶1 molar ratio and incubated for another 10 mins at 25°C. The beads were washed three times with the buffer and then resuspended in 25 µl of 4×SDS-PAGE gel loading buffer and were subjected to SDS-PAGE analysis and Coomassie blue staining.

### Fluorescence Spectroscopy

Fluorescence measurement was carried out using a spectrofluorometer, Hitachi F-7000. Fluorescence was measured at an excitation wavelength of 295 nm and an emission wavelength of 340 nm with slit widths of 2.5 nm and 5 nm for excitation and emission, respectively. All reactions were carried out at 25°C. Equilibrium titrations of *Vc*CheY3, *Vc*CheY3-Q97A/E100A, *Vc*CheY3-Q97A and *Vc*CheY3-D60A were carried out with acetyl phosphate (acP) and beryllium fluoride (BeF_3_^−^). The reactions in presence of acP were performed in a buffer containing 20 mM sodium phosphate (pH 7.5), 50 mM NaCl, and 2 mM MgCl_2_ and the same were 50 mM Tris-Cl (pH 7.5), 150 mM NaCl and 5 mM MgCl_2_ for BeF_3_^−^. For all proteins the final concentrations were 1 µM, BeF_3_^−^ concentrations varies from 0 to 400 µM and the concentrations varies from 0 to 6 mM for acP. The fluorescence values were corrected for dilution. *Km* was determined as described previously by Lukat et al (1992) [23]. Acetyl phosphate and BeF_3_^−^ concentrations were plotted versus (*I_o_* − *I*)/(*I* − *I*_inf_), where *I_o_* is initial fluorescence intensity, *I* is the intensity at the corresponding acetyl phosphate concentration, and *I*_inf_ is the intensity at the saturating concentration. From the plot, the reciprocal of the slope of the line corresponds to the *K_m_* value. According to proposed reaction scheme [23], [24], shown as follows, *K_m_* = Ks. *k3/k2*.
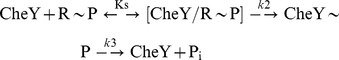
(1)


Where Ks is the equilibrium dissociation constant between CheY and acetyl phosphate (the phosphor-donor, R∼P) and *k*2 and *k*3 are the phosphorylation and dephosphorylation rate constants, respectively.

If it is assumed that the observed quenching is a direct effect of the reduced quantum yield of phospho-CheY relative to that of CheY, the steady-state fluorescence at a given concentration of phospho-donor may be related to the kinetic parameters of the reaction scheme (Eq.1), where (*I_o_−I)/(I−I_inf_*) = ([R∼P]*k2*)/(*k3*K_S_). All experimental data points were fitted by linear fit analysis using Microsoft EXCEL and Origin 8.

### Crystallization and Data Collection

Crystallization data of *Vc*CheY3 [21] and *Vc*CheY4 [22] have been published earlier. Briefly, crystals of *Vc*CheY3 that grew in low-salt condition using 5% (w/v) PEG 6000 in 0.1 M Tris–HCl pH 8.0 as precipitant, belong to space group R3 and diffracted to a resolution of 1.67 Å. Crystals of *Vc*CheY3 were also grown in the presence of Mg^2+^ in a similar condition which diffracted up to 2.2 Å. *Vc*CheY4 crystals grew in AMS at two different pH conditions. In the high-pH condition, hexagonal-shaped crystals were obtained using 0.8 M ammonium sulfate, 0.1 M Bicine pH 9.0, 4% glycerol as precipitant. In the low-pH condition, cube-shaped crystals were obtained using 0.8 M ammonium sulfate, 0.1 M citrate, 4% glycerol as precipitant. The low-pH and high-pH condition crystals were diffracted upto 1.67 Å and 1.9 Å with the space group *C2* and *P3_2_21* respectively.

Crystals of *Vc*CheY3-Q97A mutant grew in a drop consisting of 2 µl protein (6 mg/ml) solution and an equal volume of precipitant containing 1.6 M ammonium sulfate, 0.1 M Tris, pH 8.0. Cube-shaped crystals of *Vc*CheY3-Q97A belonging to space group R3 diffracted to a resolution of 2.4 Å.

Activated *Vc*CheY3 were prepared by mixing 20 µl of protein (6 mg/ml) solution with 5 µM of BeF_3_ solution and incubated for 5 minutes on ice. Crystals of activated *Vc*CheY3 were grown in a drop contains 2 µl of above mixture and equal volume of precipitant solution consisting of 10% (w/v) PEG 6000 in 0.1 M Tris–HCl pH 8.0 and equilibrated for 7 day against 20% (w/v) PEG 6000 in 0.1 M Tris–HCl pH 8.0. Activated *Vc*CheY3 crystals, after brief soaking in cryoprotectant solution containing 1 µM of BeF_3_−, diffracted upto 2.1 Å with the space group R3.

For data collection, crystals were fished out from the crystallization drops using nylon loop, briefly soaked in cryoprotectant solution and flash-cooled in a stream of nitrogen (Oxford Cryosystems) at 100 K. The diffraction data sets were collected using a MAR Research image-plate detector of diameter 345 mm and Cu K_α_ radiation generated by a Bruker–Nonius FR591 rotating-anode generator equipped with Osmic Max Flux confocal optics and operated at 50 kV and 70 mA. Data were processed and scaled using AUTOMAR (http://www.marresearch.com/automar/run.html). Data-collection and processing statistics are given in Table 1.

**Table 1:** Data collection and processing statistics.

	*Vc*CheY3 Mg^2+^ bound	*Vc*CheY3-Q97A	*Vc*CheY3-BeF_3_^−^
Space group	*R3*	*R3*	*R3*
Unit-cell parameters (Å )	a = b = 67.48, c = 74.46	a = b = 65.858, c = 65.039	a = b = 67.320, c = 72.660
Oscillation range (°)	0.5	0.5	0.5
Number of images	92	138	88
Maximum resolution (Å )	30.0–2.2	30.0–2.8	30.0–2.1
No. of molecules per ASU	1	1	1
Mathews coefficient (V_M_; Å^3^ Da^−1^)	2.23	1.86	2.19
Solvent content (%)	44.9	33.77	43.86
No. of observations	16597	9361	10428
No. of unique reflections	6341	4141	7391
Mosaicity (°)	1.59	0.5	0.35
Completeness (%)	98.9(100)	97.9(98.8)	94.3(92.2)
R_merge_† (%)	8.45(44.3)	7.39 (27.80)	3.12(22.47)
Average I/σ(I)	7.5(2.7)	5.3(2.0)	6.5(2.0)

### Structure Determination and Refinement

The structures of wild type *Vc*CheY3, *Vc*CheY4, *Vc*CheY3-Q97A and activated *Vc*CheY3 (*Vc*CheY3-BeF_3_^−^) were solved by molecular replacement using MOLREP of CCP4 suite [25]. Packing considerations indicated the presence of one molecule in the asymmetric unit for all the structures.

The wild type *Vc*CheY3 structure in its Ca^2+^ bound form was solved by using the coordinates of the *Salmonella* CheY (PDBID: 2 CHE) as template. The structure was refined by alternating cycles of model building and refinement using ‘O’ and CNS [26], [27] to a final *R*_cryst_ and *R_free_* values of 20.2% and 22.9% respectively. The poly-ala model of *Vc*CheY3 was used as search model for *Vc*CheY4 (low pH) and the refined structure of *Vc*CheY4 (low pH) was used as search model to determine the structure high pH CheY4 (*Vc*CheY4^free^). Low pH *Vc*CheY4 (*Vc*CheY4^sulf^) was refined to *R_cryst_* 21.8% and *R_free_* 24.6% and *Vc*CheY4^free^ was refined to *R_cryst_* 22.5% and *R_free_* 26.0%. *Vc*CheY3-BeF_3_^−^ structure was solved by using the coordinates of *E. coli* activated CheY i.e. *Ec*CheY-BeF_3_^−^ (PDB code: 1F4V) as the search model. Strong electron density of beryllofluoride was found close to the active-site residue D60. The structure was refined upto *R_cryst_* of 23.1% and *R_free_* of 24.3% by several rounds of refinements and manual rebuilding by using the programs *CNS* [27] and *COOT* [28], respectively. The structure of *Vc*CheY3-Q97A was solved using *Vc*CheY3 as template and refined by the similar protocol to *R_cryst_* of 22.5% and *R_free_* of 25.2%. The structure of Mg^2+^ bound *Vc*CheY3 was also solved using *Vc*CheY3 (Ca^2+^ bound) as template after removing the coordinates of Ca^2+^ and waters and refined by the similar protocol to *R_cryst_* of 20.0% and *R_free_* of 22.5%. Details of the refinement parameters for all the structures along with the geometric parameters determined by PROCHECK [29] are given in Table 2.

**Table 2:** Refinement statistics.

	*Vc*CheY3 Ca^2+^bound	*Vc*CheY3 Mg^2+^bound	*Vc*CheY3-Q97A	*Vc*CheY3-BeF_3_^−^	*Vc*CheY4^sulf^	*Vc*CheY4^free^
R_cryst_ (%)^a^	20.2	20.0	22.5	23.1	21.8	22.5
R_free_ (%)^b^	22.9	22.5	25.2	24.3	24.6	26.0
r.m.s.d bond (Å)	0.005	0.016	0.019	0.012	0.006	0.009
r.m.s.d angle (°)	1.3	1.6	2.17	1.6	1.3	1.59
No. of waters	176	94	93	107	114	144
*B*-factors (Å^2^)	19.05	25.69	48.194	27.350	19.215	46.52
***Ramachandran plot (%)*** c						
Most favored(%)	97.5	95.1	97.5	92.7	98.3	94.9
Allowed(%)	2.5	4.8	2.5	5.7	1.7	34.2
Disallowed(%)	0.0	0.0	0.0	1.6	0.0	0.9
PDB code	3TO5	4LX8	4HNQ	4HNS	4H60	4HNR

† *R*merge = ∑hkl∑i|*I*hkl-〈*I*hkl〉|/∑hkl∑i(*I*hkl), where *I*hkl is the intensity of an individual reflection and 〈*I*_hkl_〉 is the average intensity over symmetry equivalents.

a R_cryst_ = ∑_hkl_|F_obs_ F_calcd_|/∑_hkl_F_obs_, where F_obs_ and F_calcd_ are the observed and calculated structure factor amplitudes, respectively.

b *R*_free_ is the equivalent of *R*-factor, calculated for a randomly chosen set of the reflections (5%) that were omitted throughout the refinement process. V_M_ is the partial specific volume.

c As defined by PROCHECK.

### Calculation of Normalized B Factor

Since *Vc*CheY4^free^ and *Vc*CheY4^sulf^ crystals grew in different space groups and their diffraction resolutions are different, to compare their B factors we have plotted their normalized B-factor or B′-factor. Crystallographic B-factors of proteins determined even at high resolutions show large variations from one structure to another but the B-factors expressed in units of standard deviation about their mean value (normalized B-factor or B′-factor) shows consistent behaviour [30]–[32]. The equation used by us to calculate the normalized B-factor is B′ = B-<B>/σ<B>*;* where <B> is the average B value for the whole molecule based on Cα atoms and σ<B> is the standard deviation of the B values.

## Results

### Overall Structures of VcCheY3 and VcCheY4

As expected, both *Vc*CheY3 and *Vc*CheY4 possess (β/α)_5_ fold (Figure 1 b, c) typical of the response regulators. Structure of *Vc*CheY3 in free state superposes on *S. typhimurium* CheY (*St*CheY; PDB code: 2 CHE) with a root mean square deviation (rmsd) of 0.4 Å (for 108 Ca atoms) (Figure 1b). *Vc*CheY4 was crystallized in two different states; one is in free state with no ligand attached (*Vc*CheY4^free^) and another with a sulfate and a Ca^2+^ ion bound at the active site (*Vc*CheY4^sulf^). Interestingly, when *Vc*CheY4^free^ is superposed on *Vc*CheY4^sulf^ significant differences are observed at the active site, together with helix α4, β4α4 loop and β3α3 loop (Figure 1c). Since *Vc*CheY4^free^ and *Vc*CheY4^sulf^ were crystallized in different space groups, we have checked the probable influence of the crystal packing on the observed structural differences. Our packing analysis suggests that, in either case, these regions are rather loosely packed and their conformations are not influenced by crystal packing. *Vc*CheY4, in either state, is significantly different from that of *Vc*CheY3 (Figure 1b) and superposition of *Vc*CheY4^free^ and *Vc*CheY4^sulf^ on *Vc*CheY3 produces rmsd values of 1.4 Å and 1.2 Å respectively. *Vc*CheY4, in either state, differs from *Vc*CheY3 mainly in the α1, α5, α4 regions and in the β3α3 loop (Figure 1b). It is to be noted that α1 and α5 were implicated previously in CheA and FliM binding respectively [33].

**Figure 2:**
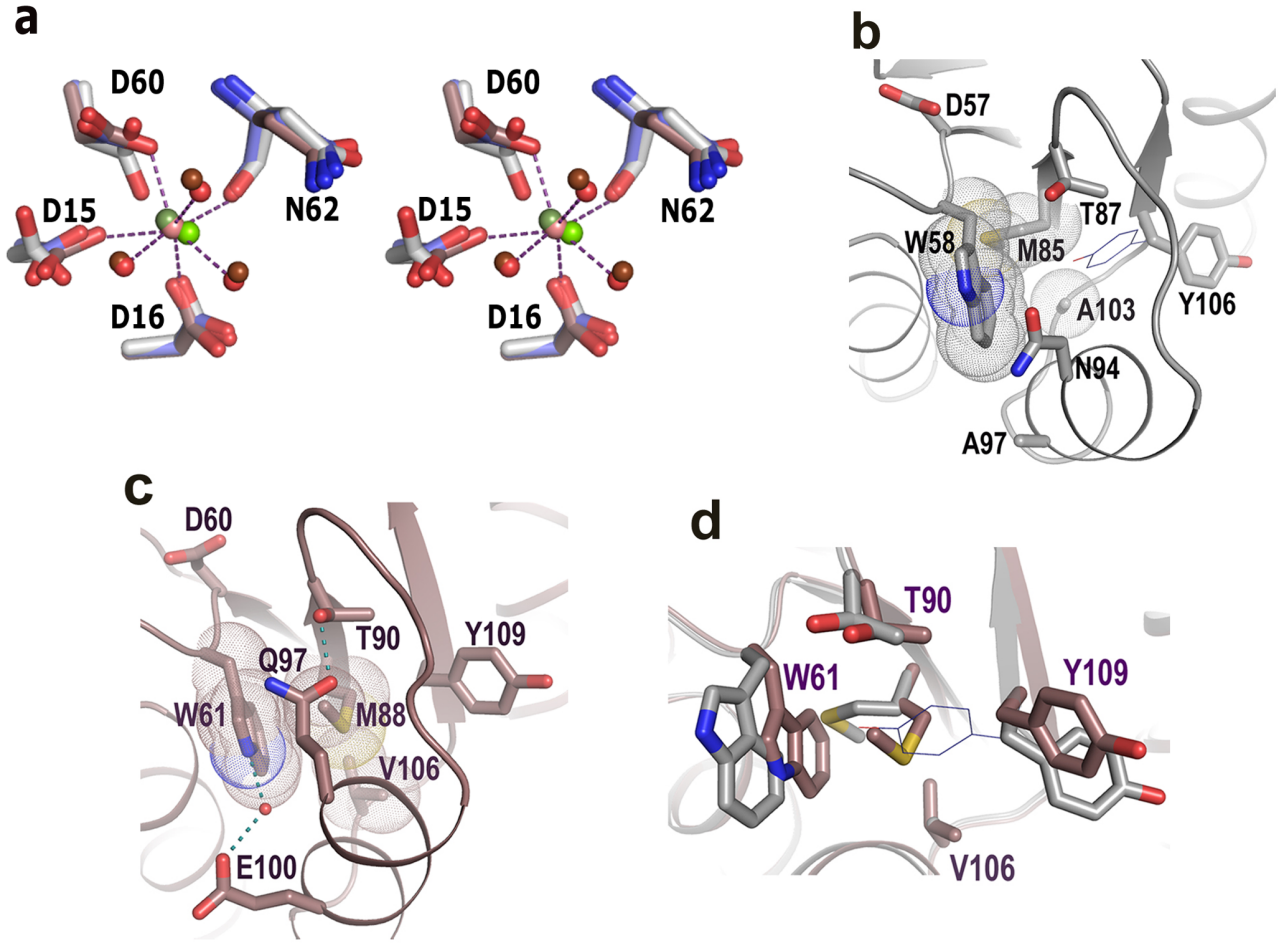
Metal binding and conformational barrier in *Vc*CheY3. (a) Stereo representation to compare the Ca^2+^ and Mg^2+^ binding at the active site of *Vc*CheY3 (violet) with the Mg^+2^ binding of *St*CheY3 (grey). Ca^2+^ bound to *Vc*CheY3 is shown as pink sphere, Mg^2+^ bound to *Vc*CheY3 is shown as dark green sphere and Mg^2+^ bound to *St*CheY3 is shown as light green sphere. Waters bound to of Ca^2+^ and Mg^2+^ are shown as light and dark red spheres respectively. Only the hydrogen bonds, observed in Ca^2+^ bound *Vc*CheY3 are shown for clarity; (b) preformed pocket for the ‘in’ position for Y106 in *Ec*CheY (thin line), coordinates for the ‘in’ position of Y106 is taken from the activated *Ec*CheY structure (PDB code:1F4V), (c) The hydrophobic packing of W61, M88, V106, hydrogen bond between T90 and Q97, and water mediated hydrogen bond between E100 and W61 that make a conformational barrier in *Vc*CheY3, (d) superposition of ‘b’ and ‘c’ showing the buried conformation of W61 and its packing with M88 in *Vc*CheY3 (violet) compared to *St*CheY (grey), ‘in’ position of Y109 (thin line) makes clashes with *Vc*CheY3 residues,

We have solved the structures of *Vc*CheY3 in Ca^2+^ and Mg^2+^ bound states to the resolutions of 1.67 Å (Figure S1a) and 2.2 Å (Figure S1b) respectively. The location of the Ca^2+^ (or Mg^2+^) ion in *Vc*CheY3 is similar to that of Mg^2+^ ion in *St*CheY. The Ca^2+^ (or Mg^2+^) of *Vc*CheY3 is heptacoordinated where four coordinations occur with protein atoms and three with water molecules (Figure 2a). In contrast to that, the Mg^2+^ of *St*CheY is hexacoordinated. Although D12 of *St*CheY is not coordinated to Mg^2+^, D15 of *Vc*CheY3 that corresponds to D12 of *St*CheY, coordinates with the metal ion (Figure 2a). Except this residue the disposition of the side chains of the other residues that coordinate with the metal ion are more or less similar in these structures (Figure 2a). The average coordination distance between Ca^2+^ and the protein atoms is about 2.4 Å while this is of about 2.1 Å in case of Mg^2+^ which is due to the size difference of the ions.

### Identification of a Conformational Barrier Towards Activation of VcCheY3

In *St*CheY or *Ec*CheY, upon phosphorylation at D57, a series of structural changes occur near the active site. T87 along with β4α4 loop moves toward the active site and stabilizes the bound phosphate through hydrogen bonding. Y106 of β5 executes an ‘inward’ movement (shown in line in Figure 2b) with minimal conformational adjustments of W58 and M85 and that inward movement of Y106 is essential for the binding of FliM at α4-β5-α5 face of CheY. K109 and the Mg^2+^ contribute to stabilize phosphorylated D57 [34]. In the free state *St*CheY, W58 stays more on the surface (with χ1 of 174°, χ2 of ­101°) and M85 side chain adopts such a χ1 value (−155°) that together these residues leave a preformed cavity for the ‘inward’ positioning of Y106 upon activation (Figure 2b).

D60 is the site of phosphorylation in *Vc*CheY3 as it corresponds to D57 of *St*CheY (Figure 1a). Both in the Mg^2+^ and Ca^2+^ bound free state structures of *Vc*CheY3, the side chain of W61 (that corresponds to W58 of *St*CheY) is observed in a conformation, substantially different from that of *St*CheY (Figure 2c, 2d). In the free state structure of *Vc*CheY3, the side chain of W61 buries unusually deeply with a χ1 of −135° and χ2 of −133° (Figure 2c). Y109 stays in its ‘out’ position and the side chain of M88 (with χ1 of 64°, χ2 of 175°) stays between W61 and Y109, packing snugly with W61, Y109 and V106 through hydrophobic interactions (Figure 2c). This packing essentially fills up the pocket, required for the ‘inward’ positioning of Y109 upon activation (Figure 2c, 2d).

Moreover, in this inactivated structure of *Vc*CheY3, the crucial T90 of β4α4 loop (that corresponds to T87 of *St*CheY), which stabilizes the bound phosphate on D60 upon activation, is hydrogen bonded with Q97 (Figure 2c). To the best of our knowledge, this kind of interaction involving the Thr of β4α4 loop was not observed so far in any other response regulator. In *Vc*CheY3, T90 and Q97 are oriented in such a fashion that together they form a capping on the aforesaid hydrophobic packing and at the same time block the ‘out to in’ trajectory of Y109 (Figure 2d). Additionally, the side chain carboxylate group of E100 (which is Ala in *Ec*CheY or *St*CheY) forms a water mediated hydrogen bond with NE1 of W61 (Figure 2c; Figure S3a, S3b). Therefore, the hydrophobic packing of W61 with M88 and V106, together with the hydrogen bond between T90 and Q97 and the water mediated interaction between W61 and E100 seem to make a conformational barrier that may affect the process of activation in *Vc*CheY3.

### Comparison of Phosphorylation Events Through Fluorescence Spectroscopy

**Figure 3:**
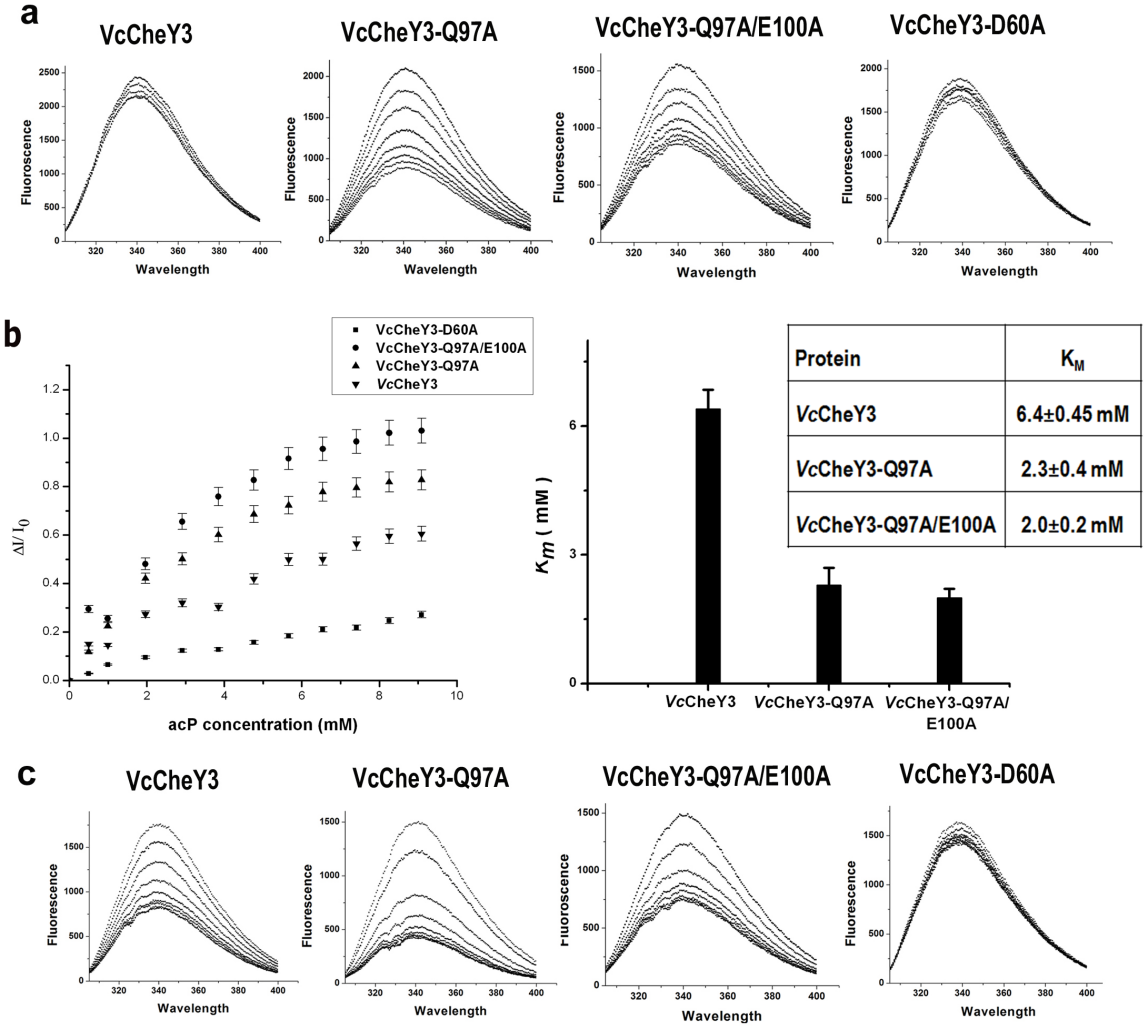
Activation of *Vc*CheY3 and its mutants, measured through fluorescence quenching. (a) Tryptophan quenching of *Vc*CheY3 and its different mutants (indicated at top of the figure) using acetyl phosphate (acP) as substrate. (b) Plot of *ΔI/I_0_* vs acP concentration (in mM) and corresponding *K_m_* values (both in graphical and numerical modes); (c) Tryptophan quenching of *Vc*CheY3 and its different mutants (indicated at top of the figure) using BeF_3_^−^ as substrate.

To investigate the contribution of the proposed ‘conformational barrier’ of *Vc*CheY3 towards its activation, we prepared three mutants *Vc*CheY3-Q97A, *Vc*CheY3-Q97A/E100A and *Vc*CheY3-D60A. Since W61 is within the Forster distance of D60, tryptophan quenching study was performed with *Vc*CheY3 and its mutants to monitor the phosphorylation event using acetyl phosphate (acP) as substrate. Interestingly, *Vc*CheY3 showed very low quenching (Figure 3a) indicating that phosphorylation at D60 does not induce any conformational change in W61 and W61 remains buried even after the treatment with acP. *Vc*CheY3-Q97A and *Vc*CheY3-Q97A/E100A, on the other hand, showed considerable quenching in the presence of acP (Figure 3a), suggesting that in the absence of the hydrogen bond between T90 and Q97 (and also in absence of E100), conformational alteration of W61 may take place more easily and it can move toward the surface of the molecule. As expected, quenching is almost negligible for the nonphosphorylatable analog *Vc*CheY3-D60A (Figure 3a). Based on these experiments we have calculated the *K_m_* (*K_m_* = Ks. *k3/k2*) values where a higher *K_m_* value implies a decrease in the binding affinity between CheY and the phosphodonor (i.e. larger Ks), a slower rate of phosphorylation of bound CheY (i.e. smaller *k2*) or a faster rate of autodephosphorylation (i.e. larger *k3*) [35]. *K_m_* value, obtained by us, was the highest for *Vc*CheY3 (6.4±0.45 mM) followed by *Vc*CheY3-Q97A (2.3±0.4 mM) and *Vc*CheY3-Q97A/E100A (2.0±0.2 mM) (Figure 3b) which are in accordance with our structural observations.

### Structure of VcCheY3-Q97A

**Figure 4:**
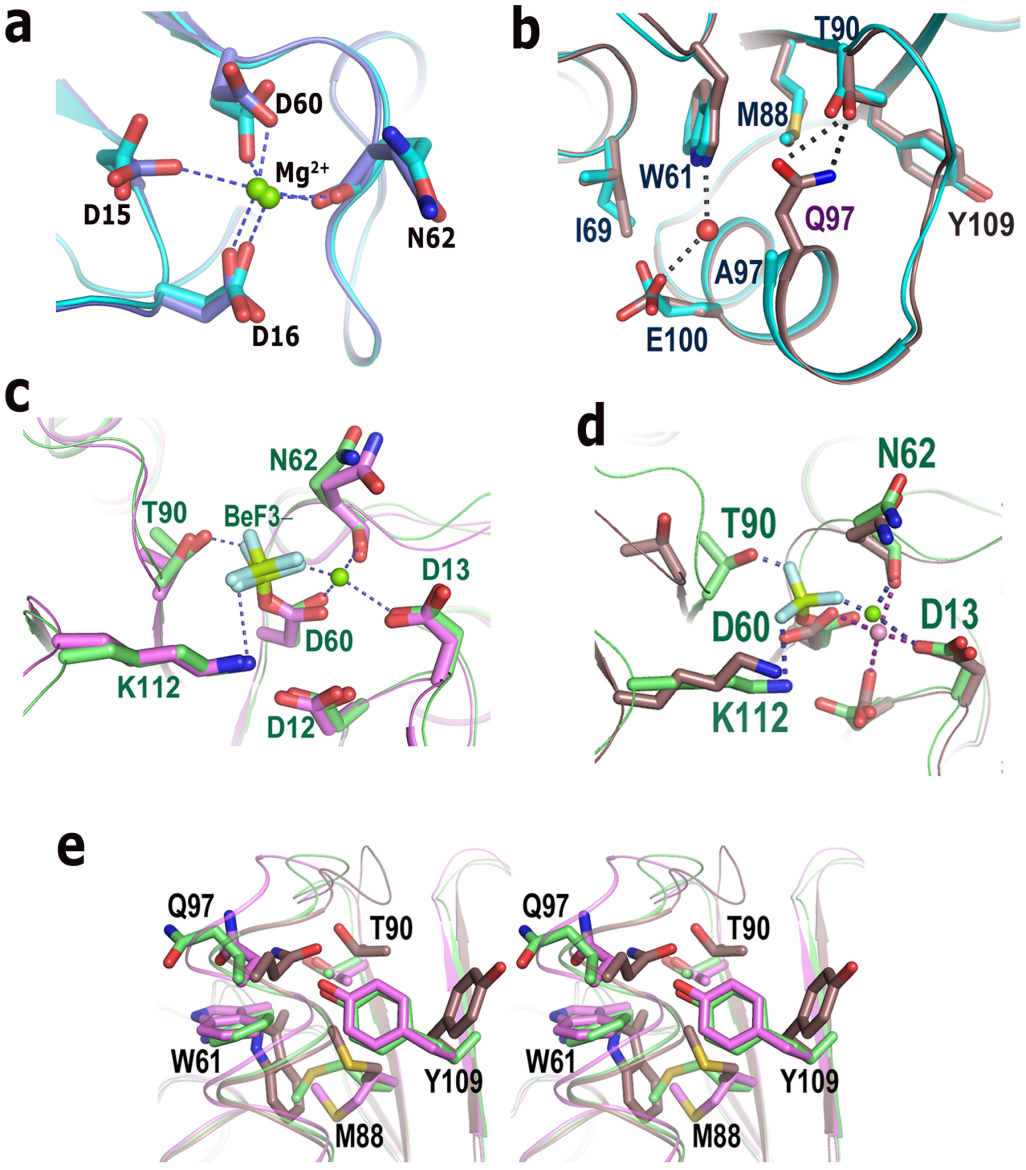
Mg^2+^ binding, activation of *Vc*CheY3 and comparison with *Ec*CheY. (a) Comparison of the Mg^2+^ binding in *Vc*CheY3 (blue) and *Vc*CheY3-Q97A (cyan); (b) superposition of *Vc*CheY3 (violet) and *Vc*CheY3-Q97A (cyan) showing that the hydrogen bond between Q97 and T90 does not directly influence the conformation of W61 and M88; (c) comparison of the active site of *Vc*CheY3-BeF_3_^−^ (green) with *Ec*CheY3-BeF_3_^−^ (magenta); (d) comparison of the active site of *Vc*CheY3-BeF_3_^−^ (green) with free state *Vc*CheY3 (violet); (e) stereoscopic representation comparing the ‘in’ position and the conformation of the neighbouring residues in *Vc*CheY3-BeF_3_^−^ (green), *Ec*CheY3-BeF_3_^−^ (magenta) with respect to *Vc*CheY3 (violet).

To investigate whether the hydrogen bond between T90 and Q97 affects the hydrophobic packing of W61, M88 and V106, we have solved the structure of *Vc*CheY3-Q97A. As expected, the overall structure of *Vc*CheY3-Q97A is almost identical to that of *Vc*CheY3 and the Mg^2+^ ion bound at the active site occupies the equivalent position to that of Mg^2+^ (or Ca^2+^) of *Vc*CheY3 (Figure 4a). Interestingly, even in the absence of the hydrogen bond between T90 and Q97, the conformation and packing of W61, M88 and V106 are found to be unaltered with respect to the wild type *Vc*CheY3 (Figure 4b). However, the water mediated hydrogen bond between W61 and E100 is not seen in this mutant. E100 is slightly reoriented here and has moved toward the CD1 atom of the adjacent I69 (Figure 4b). These observations, coupled with the quenching results, point to the fact that although the hydrophobic packing of W61, M88 and V106 is independent of the hydrogen bond between T90 and Q97 in free state, in the absence of the later interaction, reorientation of W61 and M88 occurs more smoothly upon phosphorylation.

### Structure of VcCheY3-BeF_3_^−^

Quenching data using acP (Figure 3a) clearly indicate that obtaining of stable *Vc*CheY3-P for crystallographic study is not possible. Since BeF_3_^−^ readily forms persistent activated complexes with many response regulators, regardless of the half-lives of their phosphorylated states, this is regularly used to structurally mimic the phosphorylated state of the response regulators [36]. Fluorescence quenching experiment for *Vc*CheY3 and its mutants, performed in the presence of BeF_3_^−^, showed approximately 30 fold lowering of the *K_m_* values (219.0±0.6 µM, 110.0±2.1 µM, and 96.4±1.4 µM for *Vc*CheY3, *Vc*CheY3-Q97A and *Vc*CheY3-Q97A/E100A respectively) compared to that of acP (Figure 3c). Thus, to visualize the structural changes in *Vc*CheY3 upon phosphorylation, we have activated *Vc*CheY3 using BeF_3_^−^ and solved the structure of *Vc*CheY3-BeF_3_^−^ to 2.1 Å.

The active site of *Vc*CheY3-BeF_3_^−^ largely resembles to that of *Ec*CheY-BeF_3_^−^ (PDB code: 1F4V) (Figure 4c). In *Vc*CheY3-BeF_3_^−^, BeF_3_^−^ is covalently linked with D60 and Mg^2+^ is properly poised to interact with BeF_3_^−^ (Figure 4c, 4d). To stabilize the bound BeF_3_^−^, the side chain of K112 reorients and T90 along with the β4α4 loop moves toward the active site with a conformational change, hallmark for the activation of this type of CheYs (Figure 4d). The hydrogen bond between T90 and Q97 is abolished and Q97 side chain moves away from T90 (Figure 4e). Breaking the hydrophobic packing with M88, the side chain of W61 moves toward the surface (with χ1 of −166°, χ2 of −34°) acquiring a conformation similar to that observed in *Ec*CheY-BeF_3_^−^ (Figure 4e). Under that situation, M88 occupies the space left by W61 and creates a pocket, sufficient to accommodate the ‘in’ position of Y109 which is essential for FliM binding (Figure 4e).

### Free and Sulfated Structures of VcCheY4

**Figure 5:**
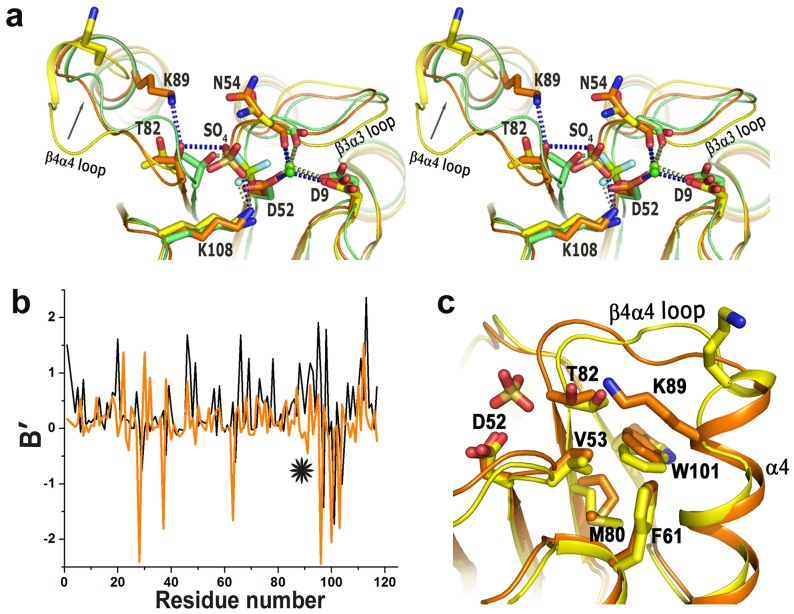
Structure of *Vc*CheY4 in free and sulphated states. (a) stereo view of the superposition of *Vc*CheY4^free^ (yellow) and *Vc*CheY4^sulf^ (orange) on activated *Vc*CheY3-BeF_3_^−^ (green) showing the location and the interactions of the sulfate ion in *Vc*CheY4^sulf^, relative movement of T82, hydrogen bond between K89 and T82 in *Vc*CheY4^sulf^ and the interactions of the metal ion with the neighbouring residues; (b) B′ plot of *Vc*CheY4^free^ (black) and *Vc*CheY4^sulf^ (orange) showing reduction of flexibility of the β4α4 loop (*) in *Vc*CheY4^sulf^; (c) superposition of *Vc*CheY4^free^ (yellow) on *Vc*CheY4^sulf^ (orange) showing the conformational difference at the β4α4 loop and packing of W101 in its exclusive ‘in’ position.

Although the overall structures of *Vc*CheY4^free^ and *Vc*CheY4^sulf^ are similar, substantial conformational differences are observed between these two, especially around the active site, in helix α4 and β4α4 loop. A Ca^2+^ ion is located at the active site of *Vc*CheY4^sulf^ which coordinates with D9, D52 and main chain carbonyl oxygen of N54 with an average coordination distance of 2.4 Å (Figure 5a; Figure S2a). A tetrahedral positive electron density was observed in the active-site pocket of *Vc*CheY4^sulf^ during refinement which was interpreted as a sulfate ion because *Vc*CheY4 was crystallized using ammonium sulfate as precipitant (Figure S2a). In contrast to that, neither a metal ion nor a sulfate ion was observed at the active site of *Vc*CheY4^free^ although both of these components were added during crystallization (Figure 5a; Figure S2b). Absence of the divalent metal ion do not cause any change in the side chain conformation of D9 and D52 compared to *Vc*CheY4^sulf^, but the carbonyl oxygen of N54 points away from the metal binding side (Figure 5a). As a result, the β3α3 loop of *Vc*CheY4^free^ takes a different conformation and moves about 3 Å away from the active site (Figure 5a). In *Vc*CheY4^free^, helix α4 is shorter and β4α4 loop is unusually longer compared to those of *Vc*CheY4^sulf^ (Figure 1c). Electron density around the β4α4 loop of *Vc*CheY4^free^ is shown in the Figure S2c. The plot of B′-values indicated that the crystallographic B-factor of the β4α4 loop is much lower in *Vc*CheY4^sulf^ compared to that of *Vc*CheY4^free^ (Figure 5b). In *Vc*CheY4^sulf^ part of the β4α4 loop is stabilized and adopts a helical structure effectively extending the length of α4 (Figure 5c) and overall, the *Vc*CheY4^sulf^ structure seems to be more compact compared to *Vc*CheY4^free^.

The location of the sulfate ion at the active site of *Vc*CheY4^sulf^ is somewhat similar to BeF_3_^−^ of *Vc*CheY3-BeF_3_^−^ (Figure 5a). T82 and K104, which are well known to stabilize the phosphoryl group in the other reported CheY structures, stabilize the sulfate ion in *Vc*CheY4^sulf^ through hydrogen bonding. A movement of about 2 Å towards the active site occurs for T82 along with the β4α4 loop (Figure 5a). Interestingly, in *Vc*CheY4^sulf^, an additional hydrogen is generated between T82 and K89 (K89 corresponds to Q97 of *Vc*CheY3) which might further contribute to the compactness of α4 in *Vc*CheY4^sulf^ (Figure 5a, 5c).

The crucial residue at β5 that acquires ‘in’ position upon activation is a Trp (W101) in case of *Vc*CheY4 and in both the structures of *Vc*CheY4 the side chain of W101 acquired ‘in’ position. In fact, this is the first structure of a naturally occurring CheY where Trp at this crucial position is observed to spontaneously occupy ‘in’ position, even without activation. In this case, W101 fits in a hydrophobic pocket made of V53, F61 and M80 (Figure 5c) and apart from making a hydrogen bond with T82, the hydrophobic part of K89 packs with W101 further contributing to the stability of *Vc*CheY4^sulf^.

### Molecular Mechanism of FliM Binding in *V. Cholerae*

**Figure 6:**
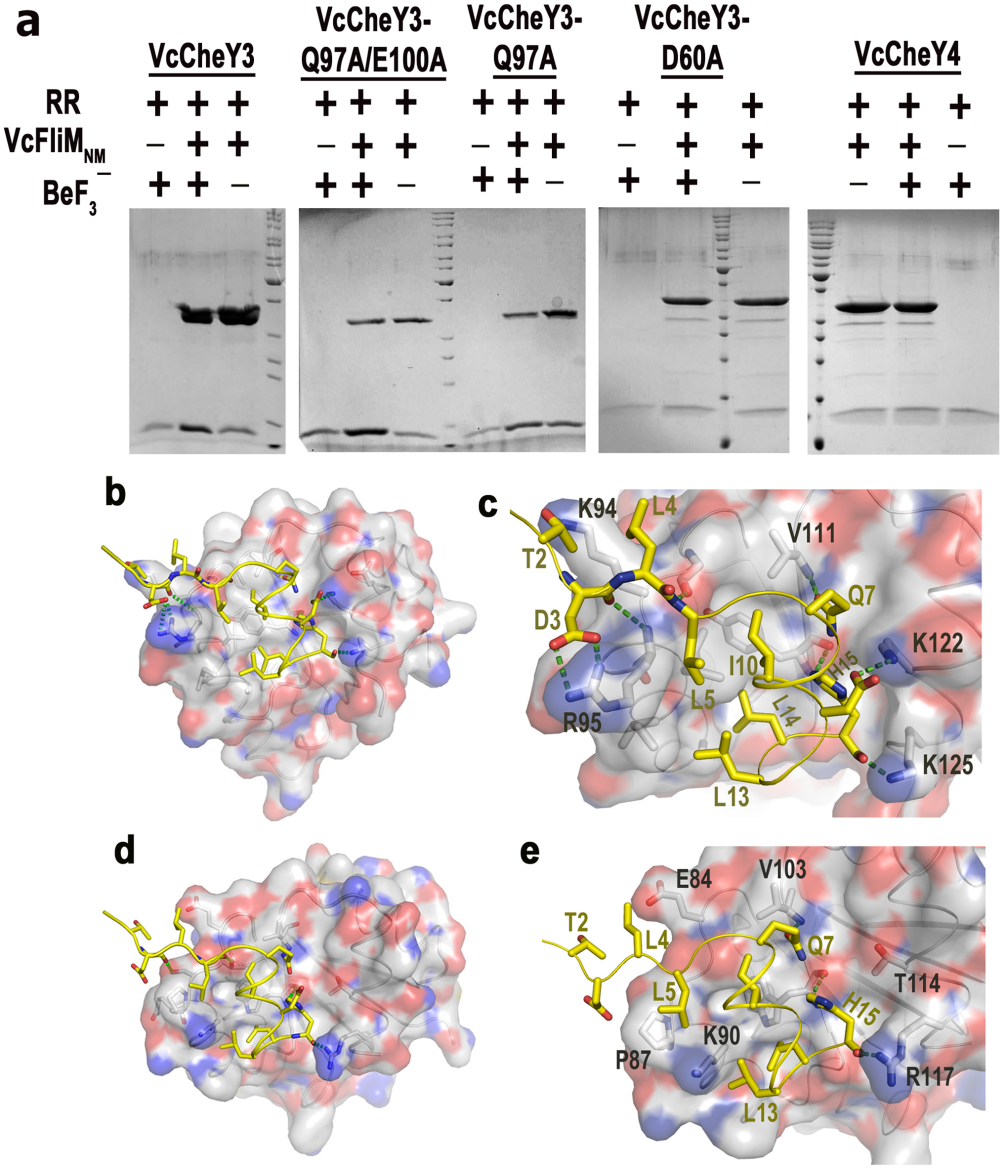
Interactions of FliM_NM_ with *Vc*CheY3, *Vc*CheY4 and *Vc*CheY3 mutants. (a) Pull-down assays of *Vc*CheY3, *Vc*CheY3-Q97A, *Vc*CheY3-Q97A/E100A, *Vc*CheY3-D60A and *Vc*CheY4 with *Vc*FliM_NM_. Purified *Vc*FliM_NM_ in 0.2 mg/ml was immobilized on pre-washed resin. *Vc*CheY3, *Vc*CheY3-D60A and *Vc*CheY4 in a 1∶1 molar ratio to *Vc*FliM_NM_ was incubated with immobilized *Vc*FliM_NM_ with or without BeF_3_^−^ at 25°C for 10 mins; (b) Docking of *Vc*FliM_N_ (16 residues) at the FliM binding face of *Vc*CheY3-BeF_3_^−^; (c) Zoomed view of (b) showing the probable interactions in detail; (d) Docking of *Vc*FliM_N_ (16 residue) at the probable FliM binding face of *Vc*CheY4^sulf^; (e) Zoomed view of (d) showing the probable interactions in detail.

To investigate the binding ability of *Vc*CheY3 and *Vc*CheY4 with *Vc*FliM, we performed an *in-vitro* pull down assay. *Vc*FliM_NM_ (a construct having the N-terminal and the middle domain of *Vc*FliM with a C-terminal 6×His-tag) was immobilized on Ni-NTA resin, which was then incubated with *Vc*CheY3, *Vc*CheY3-Q97A, *Vc*CheY3-Q97A/E100A, *Vc*CheY3-D60A and *Vc*CheY4, individually, in presence of Mg^2+^ but with or without BeF_3_^−^. Our results showed that while the activated *Vc*CheY3, *Vc*CheY3-Q97A and *Vc*CheY3-Q97A/E100A can interact with *Vc*FliM_NM_, *Vc*CheY3-D60A and *Vc*CheY4 do not show any significant interaction with *Vc*FliM_NM_ even in presence of BeF_3_^−^ and Mg^2+^ (Figure 6a). *Vc*CheY3-D60A was used as the negative control, and the experiment performed with BeF_3_^−^ and without *Vc*FliM_NM_ quantified the basal level of adherence of *Vc*CheYs in Ni-NTA agarose during experiment.

To identify the structural features of *Vc*CheY3 and *Vc*CheY4, responsible for the difference in affinity towards *Vc*FliM, it was necessary to critically analyse their FliM binding surface. To start with, we prepared a model of the N-terminal 16 peptide of *Vc*FliM_N_ by 3D-JIGSAW and *Vc*FliM_N_, thus prepared, was docked at the FliM binding face of *Vc*CheY3-BeF_3_^−^ and *Vc*CheY4^sulf^. The FliM_N_ part of the coordinates of *Ec*FliM_N_-*Ec*CheY-BeF_3_^−^ complex structure (PDB code: 1F4V) were used as a template to prepare the model of *Vc*FliM_N_ and *Ec*FliM_N_-*Ec*CheY structure as a whole was used as template for the docking. The resulting models of *Vc*FliM_N_-*Vc*CheY3-BeF_3_^−^ or *Vc*FliM_N_-*Vc*CheY4^sulf^ were then analysed to identify the structural determinants responsible for the differential FliM binding of *Vc*CheY3 and *Vc*CheY4. *Vc*FliM_N_ is observed to fit properly at the α4-β5-α5 cleft of *Vc*CheY3-BeF_3_^−^ with considerable number of hydrogen bonds and hydrophobic interactions (Figure 6b, 6c) which are comparable with those of *Ec*FliM_N_-*Ec*CheY-BeF_3_^−^ (Table 3). In contrast to that, the probable interactions of *Vc*CheY4^sulf^ with *Vc*FliM_N_ are inadequate (Figure 6d, 6e; Table 3). The FliM binding face of *Vc*CheY4^sulf^ is not compatible enough for *Vc*FliM. In *Vc*CheY4^sulf^, the space between α4 and α5 is ∼2 Å wider compared to that of *Vc*CheY3-BeF_3_^−^ which might cause a loose fit of *Vc*FliM_N_ at α4-β5-α5 face of *Vc*CheY4^sulf^. Residues T2 and D3 of *Vc*FliM_N_ are found to interact with *Vc*CheY3-BeF_3_^−^, but no such interaction is possible with *Vc*CheY4^sulf^ (Figure 6c, 6e). Furthermore, in *Vc*CheY3-BeF_3_^−^, K122 of α5 is poised to form a salt bridge with D12 of *Vc*FliM_N_, corresponding residue of *Vc*CheY4^sulf^ is T114 which is spatially away from D12 of *Vc*FliM_N_ and naturally no interaction is expected between this pair (Figure 6e). As a result, the overall interactions between *Vc*FliM_N_ and *Vc*CheY4^sulf^ are reduced significantly (Figure 6e, Table 3) supporting the observation of the pull down assay (Figure 6a).

**Table 3:** Residues of *Vc*FliM_N_ model, involved in the probable interactions with *Vc*CheY3 and *Vc*CheY4 structures, are compared with that of *Ec*FliM-*Ec*CheY structure.

*Ec*CheY3-BeF_3_^−^	*Ec*FliM_N_	*Vc*CheY3-BeF_3_^−^	*Vc*FliM_N_	*Vc*CheY4
*Polar interactions*				
K91	D3	K94 NZ	T3 OG1	–
K92 N	S4 O	R95 N	D4 O	–
		R95 NH1, NH2	D4 OD1, OD2	–
A90 O	L6, N	A93 O	L6 N	T85 O
V108 N	Q8 OE1	V111 N	Q8 OE1	V104 N
K119	D12	K122 NZ	D12 OD1	–
Y106 N	D16 OD1	Y109 O	H16 NE2	–
Y106 O	D16 N			
K122 NZ	D16 O	K125 NZ	H16 O	R117 NH1
*Hydrophobic interactions*				
I95	L6	R95, I98	L6	P87
I95, A99, Y106	I11, L14	I98, I99, Y109,V106, A102	I11, L14, L15	K90, W101

## Discussion

Unlike *E. coli* two-component chemosensory pathway that relies on a single copy of response regulator CheY, *V. cholerae* possesses four CheY homologues. Occurrence of multiple CheYs is not unusual in bacteria as these are also found in *R. sphaeroides* and *B. Burgdorferi* [5]. Recent studies have demonstrated that multiple copies of CheY play specific roles in the chemotactic signal transduction mechanisms. As for example, among the three CheYs of *B. burgdorferi* only CheY3 directly regulates motor action while the other two cannot bind to the motor and act as signal terminating phosphate sink [37]. Similarly, in *R. sphaeroides* only CheY6 can change the direction of the flagellar motor, although the others bind FliM probably to regulate the level of the phosphodonor [38], [39]. An intriguing question, therefore, arises about the role of multiple copies of CheY in *V. Cholerae*, especially of *Vc*CheY3 and *Vc*CheY4.

Together, phosphorylation at the active site Asp, hallmark movement of the Thr and the β4α4 loop toward the active site to stabilize the bound phosphate, ‘in’ positioning of the crucial hydrophobic residue of β5 and FliM binding at the α4-β5-α5 face to reverse the flagellar motion constitute the general mode of action of the chemotactic response regulators. In *Ec*CheY or *St*CheY, a preformed pocket was seen to accommodate the ‘in’ position of the crucial β5 residue Y106 upon activation (Figure 2b). In contrast to that, in *Vc*CheY3, this pocket is preoccupied by the hydrophobic packing of W61, M88 and V106 (Figure 2c). A unique hydrogen bond between T90 and Q97 additionally restricts the outward movement of W61, which is necessary to make a pocket for the ‘in’ positioning of Y109. This hydrogen bond also obstructs the movement of T90 toward the active site essentially hindering the stabilization of the phosphoryl group by T90. *Vc*CheY3 shows minimum quenching in the presence of acP which further support the hindered movement of W61 upon phosphorylation at D60 (Figure 3a). *Vc*CheY3-Q97A and *Vc*CheY3-Q97A/E100A, on the other hand, show considerable quenching in the presence of acP indicating that in the absence of the hydrogen bond between T90 and Q97, W61 can easily be reoriented toward solvent and T90 can move toward the active site to stabilize the phosphoryl group.

Higher *K_m_* value of *Vc*CheY3 compared to its mutants *Vc*CheY3-Q97A and *Vc*CheY3-Q97A/E100A further establishes the hindrance caused by the hydrogen bond between T90 and Q97 in stabilizing the acyl phosphate on D60. The lower *K_m_* values of *Vc*CheY3-Q97A and *Vc*CheY3-Q97A/E100A are due to the loss of the coupling between T90 and Q97 which facilitates the movement of T90 toward the active site and stabilize the acyl phosphate. A comparison of the *K_m_* value of *Vc*CheY3 with the CheYs from *Helicobacter pylori* or *E. coli* shows that the *K_m_* of *Vc*CheY3 is also higher than that of *Hp*CheY1 (1.07±0.31 mM) and *Ec*CheY (3.2±0.4 mM). As mentioned by Lam et al. (2010), *K_m_* increases with the increase in the ionic strength of the buffer used in the experiment [24]. While 200 mM salt was used in the experiment of *Ec*CheY, only 50 mM salt was used for *Hp*CheY1and *Vc*CheY3 (and its mutants). Since our experimental condition is same as that of *Hp*CheY1, we can clearly infer that the *K_m_* value of *Vc*CheY3 is about six fold higher than that of *Hp*CheY1.

As mentioned earlier, a higher *K_m_* (*K_m_* = Ks. *k3/k2*) implies a decrease in the binding affinity between CheY and the phosphodonor (larger Ks), a slower rate of phosphorylation of CheY (smaller *k2*) or a faster rate of autodephosphorylation (larger k3) [35]. The high *K_m_* value of *Vc*CheY3 implies that either its phosphorylation occurs slowly or it has a higher rate of autodephosphorylation. Based on the swarming assay and swimming behaviour Hyakutake et al, (2005) reported that only the *Vc*CheY3 directly switches the flagellar rotation [14]. Our pull down assay shows that *Vc*CheY3 and its mutants *Vc*CheY3-Q97A and *Vc*CheY3-Q97A/E100A bind *Vc*FliM_NM_ efficiently in the presence of BeF_3_^−^ and Mg^2+^. Docking results suggest that *Vc*FliM_N_ can fit properly at the α4-β5-α5 face of the activated *Vc*CheY3 with significant number of hydrogen bonding and hydrophobic interactions (Figure 6a, 6b; Table 3). Moreover, sequence comparison of *Vc*CheY3 with *Ec*CheY or *St*CheY denotes that the crucial residues implicated in binding the kinase CheA are conserved in *Vc*CheY3 (Figure 1a). These observations indicate that although *Vc*CheY3 has all the requisites for the phosphorylation, stabilization of the acyl phosphate is hindered due to the obstructed movement of T90 towards the active site. Lesser stabilization of the bound phosphate might be implicated in enhanced autodephosphorylation (larger *k3*) for *Vc*CheY3, effectively causing lower rate of activation which is reflected in its higher *K_m_* value. The conformational barrier of *Vc*CheY3, therefore, acts as a molecular switch to control the level of *Vc*CheY3-P. Elevated temperature and/or adequacy of phosphate pool might break the barrier of the free-state *Vc*CheY3 and flip it to the phosphorylated state for FliM binding.

Two distinct conformations, differing at helix α4 and the crucial β4α4 loop, are observed for *Vc*CheY4. Among these two structures, *Vc*CheY4^sulf^ possesses a bound sulfate ion near the active site which occupies a position similar to the BeF_3_^−^ of *St*CheY-BeF_3_^−^ and *Vc*CheY3-BeF_3_^−^ (Figure 5a). A bound sulfate ion was also observed in *Hp*CheY1 structure (PDB code: 3GWG) where that sulfate ion caused conformational changes similar to the activated structure [24]. However, in *Hp*CheY1, along with the conventional conformational changes, an unusual orientation of D53 was observed [24]. In *Vc*CheY4^sulf^, the sulfate ion did not alter the side chain conformation of catalytic D52 but stayed very close (∼2.5 Å) to it (Figure 5a). Since *Vc*CheY4^sulf^ was crystallized at pH 4.0, at this pH D52 might be protonated allowing the sulfate ion to come to its close vicinity. In *Vc*CheY4^sulf^, the sulfate ion is properly coordinated with the Ca^2+^ ion and is stabilized through the interactions with T82 and K104 (Figure 5a). Considering the compactness of the *Vc*CheY4^sulf^ structure having a shorter β4α4 loop with low B-factors, long α4 helix, movement of T82 and β4α4 loop to stabilize the sulfate ion and additional hydrogen bond between T82 and K89, it can be said that *Vc*CheY4 has a strong tendency to be phosphorylated in the presence of a divalent metal ion and the phosphorylated state is more stable compared to its free state.

Despite the fact that the crucial β5 residue W101 of *Vc*CheY4 consistently acquires ‘in’ position, *Vc*CheY4 fails to interact with *Vc*FliM_NM_ (Figure 6a). Through mutagenesis and structure-function studies Matsumura and collaborators showed that substitution of Y106 of *Ec*CheY with tryptophan (Y106W) produces a phosphorylation-dependent, hyperactive mutant that generates mainly clockwise rotational bias upon interacting with FliM [40]. In contrast to that, despite the consistent ‘in’ position of W101, *Vc*CheY4 does not interact with *Vc*FliM, as the N terminal part of *Vc*FliM does not fit at the α4-β5-α5 face of *Vc*CheY4 because of their spatial and electrostatic incompatibility (Table 3, Figure 6e). This apparent contradiction suggest that FliM binding by CheY is not just influenced by the ‘in’ positioning of the β5 hydrophoc residue but the spatial and electrostatic compatibility of the α4-β5-α5 face of CheY with the N-terminal part of FliM plays a vital role in this process. Since, CheZ and FliM share a common face of CheY for binding with similar mode of interactions [41], *Vc*CheY4 is expected not to interact efficiently with CheZ as well. This observation corroborates with the fact that no *cheZ* is found in the cluster III where *cheY4* is located. Since *Vc*CheY4 can be phosphorylated but cannot bind FliM and probably not CheZ as well, *Vc*CheY4 might act as phosphate sink or it might induce the expression of some other genes upon phosphorylation which can indirectly modulate flagellar action and/or virulence.

*Vc*CheY4 was seen to slightly enhance the spreading of an *E. coli cheZ* mutant in semisolid agar and based on that Hyakutake *et al* proposed that *Vc*CheY4 can affect chemotaxis by removing a phosphoryl group from *Vc*CheY3 [14]. Our observations intend us to hypothesise that if a phosphate pull is shared by *Vc*CheY3 and *Vc*CheY4 then *Vc*CheY4 can cause a phosphate depleted situation for *Vc*CheY3, as phosphorylated state of *Vc*CheY4 is more stable compared to its unphosphorylated state, which is other way round for *Vc*CheY3. Alternatively, in a phosphate depleted situation, additional energy might help phosphorylated *Vc*CheY4 to release the phosphoryl group through conformation dependent autodephosphorylation, as proposed by Pazy et al., 2009 [42] based on their observations of the mutant *Ec*CheY.

## Supporting Information

Figure S1 **Metal binding in** ***Vc*** **CheY3.** (a) Electron density maps (2F_o_-F_c_) around the active site of *Vc*CheY3 contoured at 1.2 σ level, Ca^2+^ is shown in pink sphere and water molecules as red dots. Ca^2+^ binding residues are labelled; (b) Electron density maps (2F_o_-F_c_) around the active site of *Vc*CheY3 contoured at 1.0 σ level, Mg^2+^ is shown as white star and waters are shown in red stars. Mg^2+^ binding residues are labelled.
(DOCX)

Figure S2 **Electron density map of** ***Vc*** **CheY4.** Electron density map (2F_o_-F_c_) contoured at 1.0 σ level (a) around the active site of *Vc*CheY4^sulf^ in stereo, (b) around the active site of *Vc*CheY4^free^, (c) around the β4α4 loop of *Vc*CheY4^free^.
(DOCX)

Figure S3 **Interaction of W61 with E100.** (a) Electron density map (2F_o_-F_c_) contoured at 1.0 σ level around the water molecule that connects W61, M88, E100 along with the water molecule in Ca^2+^ bound *Vc*CheY3; (b) Water mediated interaction of W61 with E100 in Mg^2+^ bound *Vc*CheY3.
(DOCX)

Supporting Information S1 (PDF)
