# Supplementary figures and images for: Conformational Barrier of CheY3 and Inability of CheY4 to Bind FliM Control the Flagellar Motor Action in Vibrio cholerae
Source: PLoS One. 2013 Sep 16;8(9):e73923. doi: 10.1371/journal.pone.0073923 (PMC3774744; doi:10.1371/journal.pone.0073923)

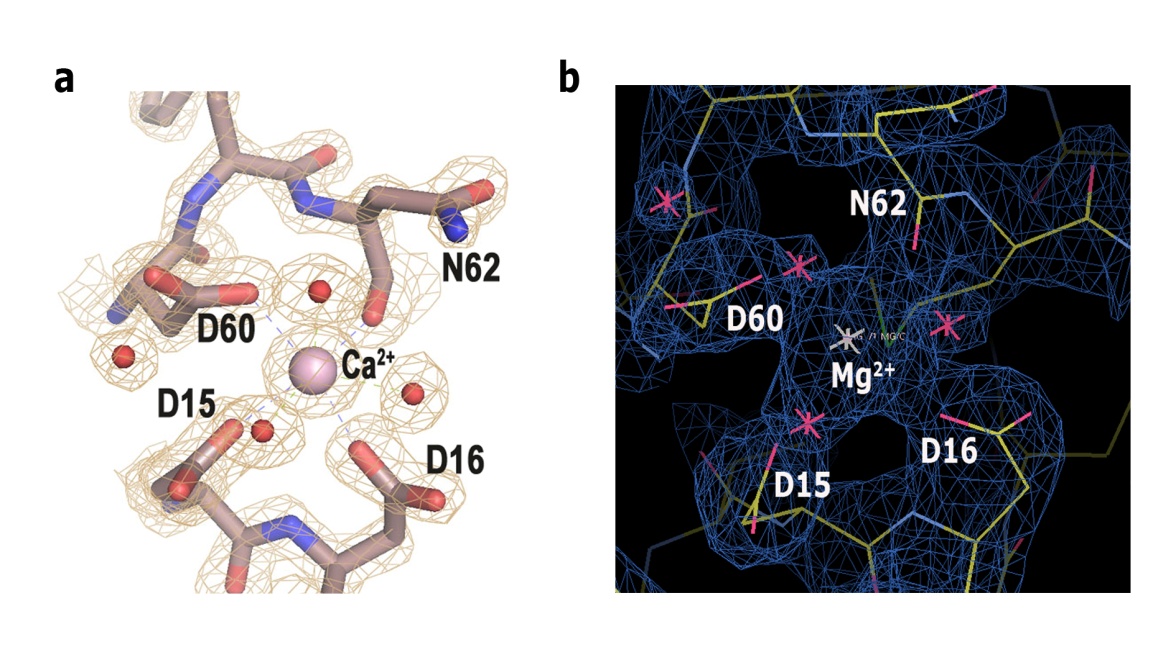

Supplement: Figure S1 — Metal binding in Vc CheY3. (a) Electron density maps (2Fo-Fc) around the active site of VcCheY3 contoured at 1.2 σ level, Ca2+ is shown in pink sphere and water molecules as red dots. Ca2+ binding residues are labelled; (b) Electron density maps (2Fo-Fc) around the active site of VcCheY3 contoured at 1.0 σ level, Mg2+ is shown as white star and waters are shown in red stars. Mg2+ binding residues are labelled. (DOCX) [file pone.0073923.s001.docx]

**(a)**


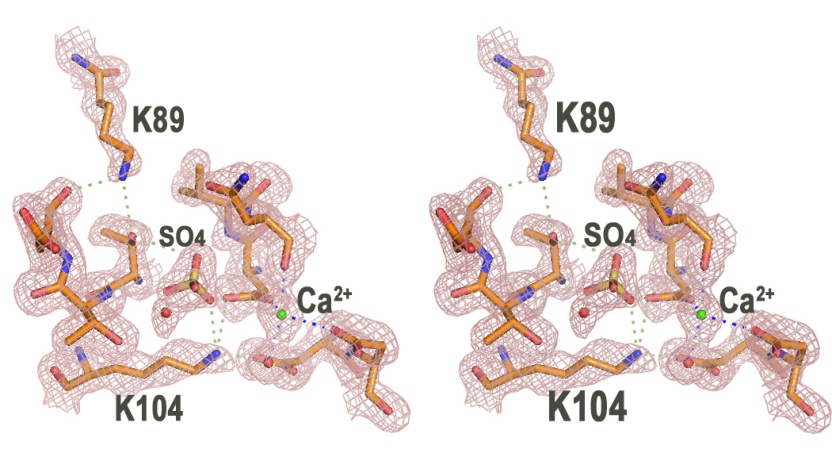


**(b)**


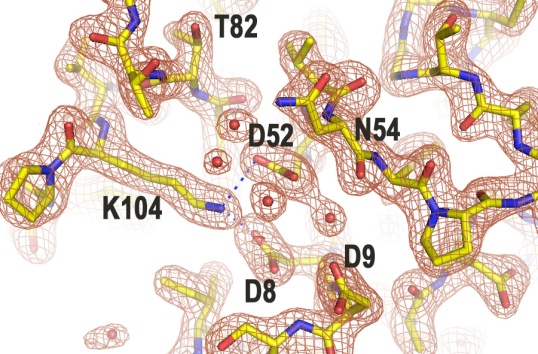


**(c)**


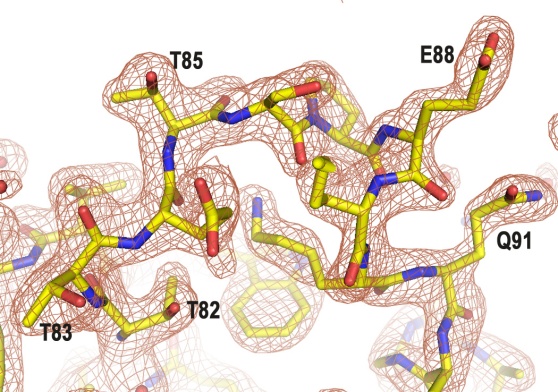

Supplement: Figure S2 — Electron density map of Vc CheY4. Electron density map (2Fo-Fc) contoured at 1.0 σ level (a) around the active site of VcCheY4sulf in stereo, (b) around the active site of VcCheY4free, (c) around the β4α4 loop of VcCheY4free. (DOCX) [file pone.0073923.s002.docx]

**a) b)**

**
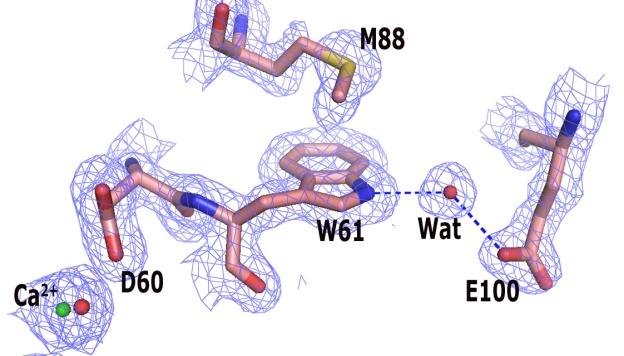

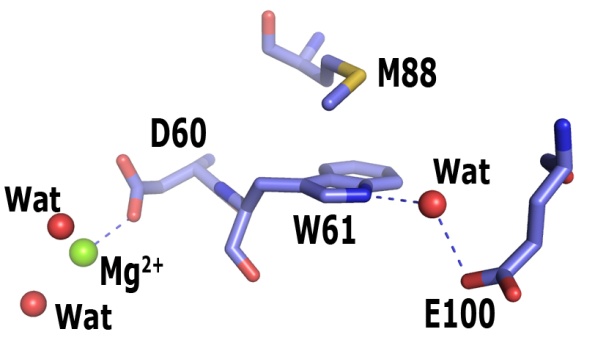
**

Supplement: Figure S3 — Interaction of W61 with E100. (a) Electron density map (2Fo-Fc) contoured at 1.0 σ level around the water molecule that connects W61, M88, E100 along with the water molecule in Ca2+ bound VcCheY3; (b) Water mediated interaction of W61 with E100 in Mg2+ bound VcCheY3. (DOCX) [file pone.0073923.s003.docx]
