# Supplementary material for: Conformational Barrier of CheY3 and Inability of CheY4 to Bind FliM Control the Flagellar Motor Action in Vibrio cholerae
Source: PLoS One. 2013 Sep 16;8(9):e73923. doi: 10.1371/journal.pone.0073923 (PMC3774744; doi:10.1371/journal.pone.0073923)
Supplement: Supporting Information S1 — (PDF) [file pone.0073923.s004.pdf]

wwPDB Validation Report

TITLE: HIGH RESOLUTION STRUCTURE OF CHEY3 FROM VIBRIO CHOLERAE
AUTHOR(S): J.DASGUPTA,U.SEN,M.BISWAS

UNOFFICIAL VALIDATION REPORT

Summary of PDB Entry Validation

The results of the validation of this PDB entry are shown below. No major issues were found.

Sequence Validation

The reported biological sequence shows no discrepancy with UniProt sequence (code Q9KQD5).

The reported biological sequence and the sequence given in the coordinates show no discrepancy.

Ligand Chemistry

Identifier: CA
Name: CALCIUM ION
Formula: Ca

Type	Program	Version	Descriptor
SMILES	ACDLabs	10.04	[Ca+2]
SMILES CANONICAL	CACTVS	3.341	[Ca++]
SMILES	CACTVS	3.341	[Ca++]
SMILES CANONICAL	OpenEye OEToolkits	1.5.0	[Ca+2]
SMILES	OpenEye OEToolkits	1.5.0	[Ca+2]
InChI	InChI	1.03	InChI=1S/Ca/q+2
InChIKey	InChI	1.03	BHPQYMZQTOCNFJ-UHFFFAOYSA-N

Summary of Structure Factor Validation

RCSB PDB PROTEIN DATA BANK

Structure quality	
Average Real space R-factor (Calculated by SFCHECK, V7.02.4)	0.0583
Average Real-space correlation coefficient (Calculated by SFCHECK, V7.02.4)	0.9916
Average Occupancy-weighted avg temperature factor	20.98

Resolution	
High Resolution (Author reported)	1.65
High Resolution (Calculated by SFCHECK, V7.02.4)	1.65
Low Resolution (Author reported)	23.26
Low Resolution (Calculated by SFCHECK, V7.02.4)	23.26

Crystal data	
Space group	H 3
Total number of reflections	14876
Number of reflections used	14112

R-factors	
R-factor (Author reported)	0.202
R-factor (Calculated by SFCHECK, V7.02.4)	0.208
Free R-factor (Author reported)	0.229
Free R-factor (Calculated by SFCHECK, V7.02.4)	0.240

Wilson statistics (PHENIX, V1.6-289)	
Wilson B-factor	17.08
Wilson Scale	0.35

Padilla-Yeates statistics for twin detection (PHENIX, V1.6-289)	
Padilla-Yeates <|L|>	0.476
Padilla-Yeates <L*L>	0.309

UNOFFICIAL VALIDATION REPORT

RCSB PDB PROTEIN DATA BANK

wwPDB Validation Report

UNOFFICIAL VALIDATION REPORT

Summary of PDB Entry Validation

The results of the validation of this PDB entry are shown below. No major issues were raised during data processing.

Geometry Validation

1. **Atomic Clashes**

No issues found.

2. **Peptide Linkage**

No issues found.

3. **Covalent Geometry**

No issues found.

4. **Chirality Error**

No issues found.

5. **Phi/Psi Torsion Angles**

No issues found.

Individual Residue Outliers on Real Space R-value

No issues found.

Ligand Chemistry

Ligand chemistry has been checked against the Chemical Component Dictionary. The following is a summary.

UNOFFICIAL VALIDATION REPORT

RCSB PDB PROTEIN DATA BANK

Identifier: MG
Name: MAGNESIUM ION
Formula: Mg

Type	Program	Version	Descriptor
SMILES	ACDLabs	10.04	[Mg+2]
SMILES CANONICAL	CACTVS	3.341	[Mg++]
SMILES	CACTVS	3.341	[Mg++]
SMILES CANONICAL	OpenEye OEToolkits	1.5.0	[Mg+2]
SMILES	OpenEye OEToolkits	1.5.0	[Mg+2]
InChI	InChI	1.03	InChI=1S/Mg/q+2
InChIKey	InChI	1.03	JLVVSXFLKOJNIY-UHFFFAOYSA-N

UNOFFICIAL VALIDATION REPORT

RCSB PDB PROTEIN DATA BANK

Summary of Structure Factor Validation

UNOFFICIAL VALIDATION REPORT

Structure quality	
Average Real space R-factor (Calculated by SFCHECK, V7.02.4)	0.0839
Average Real-space correlation coefficient (Calculated by SFCHECK, V7.02.4)	0.9808
Average Occupancy-weighted avg temperature factor	25.552

Resolution	
High Resolution (Author reported)	2.20
High Resolution (Calculated by SFCHECK, V7.02.4)	2.20
High Resolution (Calculated by REFMAC, V5.7.0029)	2.200
High Resolution (Calculated by PHENIX, V1.8-1069)	2.20
Low Resolution (Author reported)	45.97
Low Resolution (Calculated by SFCHECK, V7.02.4)	24.82
Low Resolution (Calculated by REFMAC, V5.7.0029)	24.820
Low Resolution (Calculated by PHENIX, V1.8-1069)	24.82

Crystal data	
Space group	H 3
Total number of reflections	6044
Number of reflections used	5753
Completeness of data	98.83

R-factors	
R-factor (Author reported)	0.20036
R-factor (Calculated by SFCHECK, V7.02.4)	0.227
R-factor (Calculated by REFMAC, V5.7.0029)	0.1996
R-factor (Calculated by PHENIX, V1.8-1069)	0.1913
Free R-factor (Author reported)	0.22503
Free R-factor (Calculated by SFCHECK, V7.02.4)	0.244
Free R-factor (Calculated by REFMAC, V5.7.0029)	0.2200

RCSB PDB PROTEIN DATA BANK

Free R-factor (Calculated by PHENIX, V1.8-1069)	0.2166

**Wilson statistics (PHENIX, V1.8-1069)**	
Wilson B-factor	37.49
Wilson Scale	1.07

**Padilla-Yeates statistics for twin detection (PHENIX, V1.8-1069)**	
Padilla-Yeates <|L|>	0.486
Padilla-Yeates <L*L>	0.317

UNOFFICIAL VALIDATION REPORT

RCSB PDB PROTEIN DATA BANK

wwPDB Validation Report

Summary of PDB Entry Validation

The results of the validation of this PDB entry are shown below. Some major issues were found.

Geometry Validation

1. **Peptide Linkage**

The C-N bonds listed below lie outside of the accepted range for the peptide bond (1.30-1.45). The main chain geometry needs to be corrected.

```
Residue A ILE 89 and Residue A THR 90 are not properly linked:
 C-N bond distance is 1.15 A.
```

Sequence Validation

The reported biological sequence and the sequence given in the coordinates show no discrepancy.

Ligand Chemistry

Identifier: BEF
Name: BERYLLIUM TRIFLUORIDE ION
Formula: Be F3

UNOFFICIAL VALIDATION REPORT

RCSB PDB PROTEIN DATA BANK

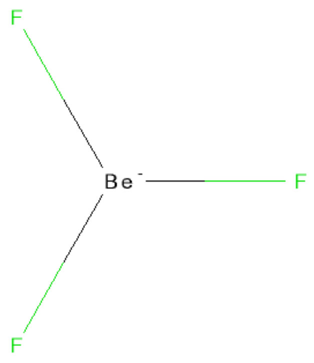

Type	Program	Version	Descriptor
SMILES	ACDLabs	10.04	F[Be-](F)F
SMILES CANONICAL	CACTVS	3.341	F[Be-](F)F
SMILES	CACTVS	3.341	F[Be-](F)F
SMILES CANONICAL	OpenEye OEToolkits	1.5.0	[Be-](F)(F)F
SMILES	OpenEye OEToolkits	1.5.0	[Be-](F)(F)F
InChI	InChI	1.03	InChI=1S/Be.3FH/h;3*1H/q+2;;;/p-3
InChIKey	InChI	1.03	OGIAHMCCNXDTIE-UHFFFAOYSA-K

Identifier: MG2
Status: Not found in chemical component dictionry

Summary of Structure Factor Validation

Structure quality	
Average Real space R-factor (Calculated by SFCHECK, V7.02.4)	0.1085
Average Real-space correlation coefficient (Calculated by SFCHECK, V7.02.4)	0.9688

UNOFFICIAL VALIDATION REPORT

RCSB PDB PROTEIN DATA BANK

Average Occupancy-weighted avg temperature factor	56.15

Resolution	
High Resolution (Author reported)	2.10
High Resolution (Calculated by SFCHECK, V7.02.4)	2.10
Low Resolution (Author reported)	24.22
Low Resolution (Calculated by SFCHECK, V7.02.4)	33.66

Crystal data	
Space group	H 3
Total number of reflections	6882
Number of reflections used	6531

R-factors	
R-factor (Author reported)	0.231
R-factor (Calculated by SFCHECK, V7.02.4)	0.250
Free R-factor (Author reported)	0.243
Free R-factor (Calculated by SFCHECK, V7.02.4)	0.274

Wilson statistics (PHENIX, V1.6-289)	
Wilson B-factor	46.21
Wilson Scale	0.51

Padilla-Yeates statistics for twin detection (PHENIX, V1.6-289)	
Padilla-Yeates <|L|>	0.494
Padilla-Yeates <L*L>	0.326

UNOFFICIAL VALIDATION REPORT

RCSB PDB PROTEIN DATA BANK

wwPDB Validation Report

UNOFFICIAL VALIDATION REPORT

Summary of PDB Entry Validation

The results of the validation of this PDB entry are shown below. No major issues were found.

Sequence Validation

The reported biological sequence and the sequence given in the coordinates show no discrepancy.

Ligand Chemistry

Identifier: MG2
Status: Not found in chemical component dictionry

Summary of Structure Factor Validation

Structure quality	
Average Real space R-factor (Calculated by SFCHECK, V7.02.4)	0.1069
Average Real-space correlation coefficient (Calculated by SFCHECK, V7.02.4)	0.9695
Average Occupancy-weighted avg temperature factor	43.16

Resolution	
High Resolution (Author reported)	2.40
High Resolution (Calculated by SFCHECK, V7.02.4)	2.20
Low Resolution (Author reported)	23.07
Low Resolution (Calculated by SFCHECK, V7.02.4)	23.07

Crystal data	
Space group	H 3
Total number of reflections	4840
Number of reflections used	4586

R-factors	
R-factor (Author reported)	0.225
R-factor (Calculated by SFCHECK, V7.02.4)	0.247
Free R-factor (Author reported)	0.252
Free R-factor (Calculated by SFCHECK, V7.02.4)	0.272

Wilson statistics (PHENIX, V1.6-289)	
Wilson B-factor	42.05
Wilson Scale	0.28

<table>
<tr><th>Padilla-Yeates statistics for twin detection (PHENIX, V1.6-289)</th><th></th></tr>
<tr><td>Padilla-Yeates <|L|></td><td>0.484</td></tr>
<tr><td>Padilla-Yeates <L*L></td><td>0.317</td></tr>
</table>

UNOFFICIAL VALIDATION REPORT

wwPDB Validation Report

UNOFFICIAL VALIDATION REPORT

Summary of PDB Entry Validation

The results of the validation of this PDB entry are shown below. No major issues were found.

Sequence Validation

The reported biological sequence and the sequence given in the coordinates show no discrepancy.

Ligand Chemistry

Identifier: CA
Name: CALCIUM ION
Formula: Ca

Type	Program	Version	Descriptor
SMILES	ACDLabs	10.04	[Ca+2]
SMILES CANONICAL	CACTVS	3.341	[Ca++]
SMILES	CACTVS	3.341	[Ca++]
SMILES CANONICAL	OpenEye OEToolkits	1.5.0	[Ca+2]
SMILES	OpenEye OEToolkits	1.5.0	[Ca+2]
InChI	InChI	1.03	InChI=1S/Ca/q+2
InChIKey	InChI	1.03	BHPQYMZQTOCNFJ-UHFFFAOYSA-N

Identifier: SO4
Name: SULFATE ION
Formula: O4 S

RCSB PDB PROTEIN DATA BANK

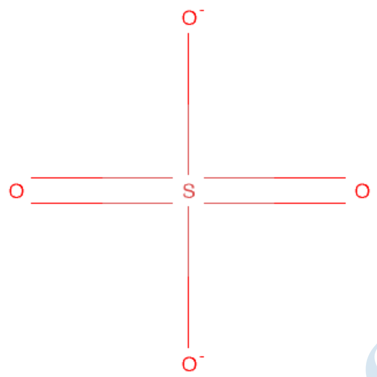

Type	Program	Version	Descriptor
SMILES	ACDLabs	10.04	[O-]S([O-])(=O)=O
SMILES CANONICAL	CACTVS	3.341	[O-][S]([O-])(=O)=O
SMILES	CACTVS	3.341	[O-][S]([O-])(=O)=O
SMILES CANONICAL	OpenEye OEToolkits	1.5.0	[O-]S(=O)(=O)[O-]
SMILES	OpenEye OEToolkits	1.5.0	[O-]S(=O)(=O)[O-]
InChI	InChI	1.03	InChI=1S/H2O4S/c1-5(2,3)4/h(H2,1,2,3,4)/p-2
InChIKey	InChI	1.03	QAOWNCQODCNURD-UHFFFAOYSA-L

Summary of Structure Factor Validation

Structure quality	
Average Real space R-factor (Calculated by SFCHECK, V7.02.4)	0.0687
Average Real-space correlation coefficient (Calculated by SFCHECK, V7.02.4)	0.9881
Average Occupancy-weighted avg temperature factor	20.32

Resolution	

UNOFFICIAL VALIDATION REPORT

High Resolution (Author reported)	1.66
High Resolution (Calculated by SFCHECK, V7.02.4)	1.62
Low Resolution (Author reported)	22.24
Low Resolution (Calculated by SFCHECK, V7.02.4)	22.24

**Crystal data**	
Space group	C 2
Total number of reflections	10882
Number of reflections used	10334

**R-factors**	
R-factor (Author reported)	0.218
R-factor (Calculated by SFCHECK, V7.02.4)	0.215
Free R-factor (Author reported)	0.246
Free R-factor (Calculated by SFCHECK, V7.02.4)	0.239

**Wilson statistics (PHENIX, V1.6-289)**	
Wilson B-factor	15.77
Wilson Scale	0.58

**Padilla-Yeates statistics for twin detection (PHENIX, V1.6-289)**	
Padilla-Yeates <|L|>	0.463
Padilla-Yeates <L*L>	0.305

UNOFFICIAL VALIDATION REPORT

wwPDB Validation Report

UNOFFICIAL VALIDATION REPORT

Summary of PDB Entry Validation

The results of the validation of this PDB entry are shown below. No major issues were found.

Sequence Validation

The reported biological sequence and the sequence given in the coordinates show no discrepancy.

Ligand Chemistry

Identifier: SO4
Name: SULFATE ION
Formula: O4 S

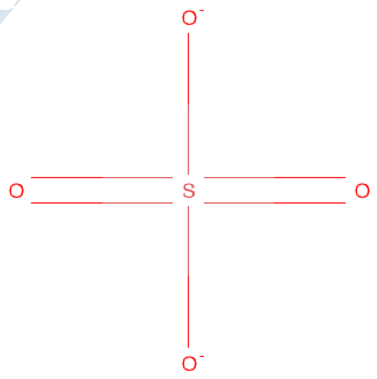

Type	Program	Version	Descriptor
SMILES	ACDLabs	10.04	[O-]S([O-])(=O)=O

SMILES CANONICAL	CACTVS	3.341	[O-][S]([O-])(=O)=O
SMILES	CACTVS	3.341	[O-][S]([O-])(=O)=O
SMILES CANONICAL	OpenEye OEToolkits	1.5.0	[O-]S(=O)(=O)[O-]
SMILES	OpenEye OEToolkits	1.5.0	[O-]S(=O)(=O)[O-]
InChI	InChI	1.03	InChI=1S/H2O4S/c1-5(2,3)4/h(H2,1,2,3,4)/p-2
InChIKey	InChI	1.03	QAOWNCQODCNURD-UHFFFAOYSA-L

Summary of Structure Factor Validation

**Structure quality**	
Average Real space R-factor (Calculated by SFCHECK, V7.02.4)	0.0886
Average Real space R-factor (Calculated by MAPMAN, V7.8.5)	0.1004
Average Real-space correlation coefficient (Calculated by SFCHECK, V7.02.4)	0.9789
Average Real-space correlation coefficient (Calculated by MAPMAN, V7.8.5)	0.9167
Average Occupancy-weighted avg temperature factor	38.43

**Resolution**	
High Resolution (Author reported)	1.90
High Resolution (Calculated by SFCHECK, V7.02.4)	1.87
High Resolution (Calculated by REFMAC, V5.5.0109)	1.867
Low Resolution (Author reported)	18.37
Low Resolution (Calculated by SFCHECK, V7.02.4)	18.36
Low Resolution (Calculated by REFMAC, V5.5.0109)	18.364

**Crystal data**	
Space group	P 32 2 1
Total number of reflections	10649
Number of reflections used	10121

UNOFFICIAL VALIDATION REPORT

RCSB PDB PROTEIN DATA BANK

R-factors	
R-factor (Author reported)	0.227
R-factor (Calculated by SFCHECK, V7.02.4)	0.239
R-factor (Calculated by REFMAC, V5.5.0109)	0.2281
Free R-factor (Author reported)	0.256
Free R-factor (Calculated by SFCHECK, V7.02.4)	0.271
Free R-factor (Calculated by REFMAC, V5.5.0109)	0.2620

Wilson statistics (PHENIX, V1.6-289)	
Wilson B-factor	34.61
Wilson Scale	0.41

Padilla-Yeates statistics for twin detection (PHENIX, V1.6-289)	
Padilla-Yeates <|L|>	0.505
Padilla-Yeates <L*L>	0.337

UNOFFICIAL VALIDATION REPORT

RCSB PDB PROTEIN DATA BANK
